# Modeling Key Strategies for Reducing Socio-Economic and Health Crisis: Perspective from COVID-19 Pandemic

**DOI:** 10.3390/ijerph192114127

**Published:** 2022-10-29

**Authors:** Sajid Ullah, Farman Ullah Khan, Vanina Adoriana Trifan, Adina Eleonora Spinu, Grigorie Sanda

**Affiliations:** 1 School of Economics and Management, Xi’an University of Technology, Xi’an 710048, China; 2 School of Management, Xi’an Jiaotong University, Xi’an 710049, China; 3 Department of Economic Disciplines, Aurel Vlaicu University of Arad, 310130 Arad, Romania

**Keywords:** socio-economic crisis, COVID-19 pandemic, containment strategies, psychological stress, interpretive structural modeling

## Abstract

The pandemic outbreak has dramatically changed every sector and walk of life. Specifically, the developing countries with scarce resources are facing unprecedented crises that further jeopardize efforts to achieve sustainable life. Considering the case of a developing country, Pakistan, this study empirically identifies the most important strategies to reduce the socio-economic and health challenges during COVID-19. Initially, the study identified 14 key strategies from the prior literature. Later, these strategies were determined with the help of the interpretive structural modeling (ISM) approach through expert suggestions. The ISM model represents seven levels of pandemic containment strategies based on their significance level. The strategies existing at the top level of ISM model are the least important, while the strategies at the bottom of hierarchy levels are highly significant. Therefore, the study results demonstrated that “strong leadership and control” and “awareness on social media” play significant roles in reducing pandemic challenges, while “promoting online purchase behavior” and “online education” are the least important strategies in tackling pandemic crisis. This study will benefit government authorities and policymakers, enabling them to focus more on significant measures in battling this ongoing crisis.

## 1. Introduction

An outbreak of pneumonia with an unknown etiology was reported at the end of December 2019 in Wuhan city (China), but it was later discovered that the cases emerged from a novel infection disease (COVID-19) that spread rapidly around the world [1]. The number of reported cases surged exponentially around the globe in a short time span, prompting the WHO to declare COVID-19 a “pandemic” on March 11, 2020 [2]. On 26 February 2020, the first case of COVID-19 was confirmed in Pakistan’s largest city, Karachi. As COVID-19 cases increases, it urges healthcare system, governments, and practitioners to invest in health infrastructure, social and economic factors [3,4,5]. 

Like many other nations, the pandemic dealt a significant blow to Pakistan’s grave economy. The small- and medium–sized enterprises of the country are facing challenges of cash flow due to the current pandemic crisis. Further, those businesses that are still open are incurring additional costs to purchase face masks, gloves, and sanitizer to protect staff health. Due to the prolonged crisis and lockdown, Pakistan’s unemployment rate jumped from 5.8% in (2017–18) to 8.1% in (2020–21) [2]. COVID-19 has transformed policymakers’ understanding into remarkable legislation that could bring favorable results to the lives of individuals, corporations, and organizations. Recent studies demonstrated that sustainable development in the pandemic could be achieved by prioritizing macro- and micro-level strategies related to the economy, society, and environment. However, the local residents need to adapt to country-based policies, challenges, and environment [6]. One of the best strategies to promote mental health and well-being is to adopt regular exercise such as gym, yoga, running. and other healthy activities. The emerging pandemic literature advocates that people with hobbies such as gardening, painting, crafting, and sewing can reduce stress and anxiety, which is very useful during pandemic [7].

Generally, epidemics not only affect the public health system but also cause socioeconomic and political crises in the society [8]; because such viral diseases may not impact everyone equally [9], it is hard to understand the impact of viral disease on diverse economic and social lives. The risk of infection depends on education, rural or urban location, occupational status, population density, and household size [10]. The poor developing nations cannot incorporate social distancing and lockdown strategies effectively due to unhealthy living conditions, large household size, and limited socio-economic support; thus, these communities are more exposed to the pandemic outbreak [11,12,13]. As a result, there is a growing need to stop or reduce these challenges by devising effective measures to improve resilient communities. From the previous pandemic literature, there are no studies which combined key socio-economic and health policies for supporting citizens during pandemic. In addition, researchers have failed to establish causal relationships between factors or events in dynamic situations by considering their cross-impacts for the identified time horizon. In fact, there is no reliable framework which has presented the pandemic containment factors in an understandable way. The presentation of such strategies is useful for policymakers, government officials in implementation, and in the same way, citizens can also receive a benefit by adopting such key factors. Because such a technique includes the best use of the resources at hand to accomplish the national goals, it is achieving consensus on the setting of priorities. Priorities must be agreed upon by all agencies due to the diversity of their resources, organizational objectives, understanding of issues, internal communication, and awareness of difficulties. In the absence of a well-defined vaccine, mathematical modeling is critical for the better understanding of epidemic dynamics and developing approaches for curbing the infectious diseases. Such a modeling approach is already proven to be effective in battling COVID-19 challenges. One of the MCDM approaches—interpretive structural modeling (ISM)—is the best technique to solve complex problems in a simple systematic way. This technique is useful because it compiles and presents important factors in systematic structured form. Thus, the main goal of this study is to fill the existing knowledge gap by identifying and prioritizing key measures with the help of mathematical modeling in the context of Pakistan’s limited resource settings. Identifying and prioritizing the key strategies for crisis management is a more effective technique to address such issues [14,15], which can be achieved with the help of the ISM technique. This type of approach usually helps in identifying and establishing links among identified factors. Therefore, this study raises certain questions which should be addressed:

(1) What are the significant strategies that can provide socio-economic and health support to citizens during the pandemic?
(2) What are the relationships among identified factors?

Based on the above queries, the study aims: 

To identify key strategies in order to reduce health, social, and economic challenges in the pandemic; 

To establish a contextual relationship among identified strategies;

To quantify the drive and dependence power of listed strategies.

The reminder of this paper is designed as follows: The emerging pandemic literature and study gap is presented, and Section 3 outlines research methodology. The subsequent sections describe results of analysis, discussion, policy implications, and the conclusion.

## 2. Literature Review

The spread of the COVID-19 pandemic has a detrimental impact on public health, national economies, and social lives. Globally, 163.7 million cases and more than 3.3 million deaths has been reported as of May 20, 2021 [16]. Many studies have assessed the effectiveness of non-pharmaceutical interventions (NPIs) to reduce the spread of COVID-19, such as avoiding gatherings, surveillance, shut downs, curfews, travel bans, and school and college closures [17,18]. While several articles have assessed the success of the measures used to prevent the spread of COVID-19, very limited work has looked at the factors that motivated these measures [19]. In a similar study, the author reviewed the experiences of a list of nations that managed the COVID-19 situation quite well. The objective is to gather knowledge and ideas that will benefit nations that might suffer in the pandemic’s initial or second-round outbreaks. The three key drivers of the effectiveness of COVID-19 policies are found to be healthcare, social protection, and general governance systems [20]. Among all previous successful pandemic containment strategies, one of the remarkable strategy is “lockdown”, while the use of social distancing also plays a supportive role in the containment of the outbreak [21,22]. However, strict enforcement of health preventive measures without socioeconomic support leads to economic unrest [23], equitable issues [24], psychological stress [25,26], depression, tension, and anxiety [27]. In this regard, it is crucial to mitigate the psychosocial stress through proper risk response and effective planning, strengthening healthcare capacity, behavioral and emotional support, and good governance with multi-sectoral coordination [28].

The COVID-19 epidemic has damaged both environmental and community health. The majority of the healthcare wastes (HCWs) are non-infectious wastes, making up the remaining 15–20%. However, when a communicable disease epidemic such as COVID-19 occurs, all HCWs that come into contact with the infected individuals become infectious. In the middle of the COVID-19 epidemic, those who were affected with the viral disease were being quarantined at home and produced hazardous trash [29]. Since the COVID-19 virus may survive longer on plastic and stainless steel, healthcare wastes (HCWs) generated during the care of COVID-19-positive patients may represent one possible pathway for infection spread [30]. To avoid disruptions in the delivery of healthcare services in the context of India, the author identifies and analyzes the determinants of resilient healthcare supply chain (HCSC) preparedness in emergency health outbreaks [31]. The health-care supply chain (HCSC) disruptions and uncertainties have increased as a result of the significant rise of COVID-19 cases in India, posing serious challenges to healthcare facilities and seriously affecting the operation of the health industry. The previous studies intend to identify inter-relationships among them in the health-care market and present a hierarchical structural model of HCSC enablers in the COVID-19 epidemic [32].

The underserved communities in Pakistan also face challenges in the pandemic, such as extreme poverty, hunger, scarce resources and opportunities, unemployment, job insecurity, and limited financial and social support [33]. Unfortunately, the South Asian countries are grappling with the shortage of quality health infrastructure to tackle the crisis, e.g., Pakistan has just 980 physicians for one million people, while many hospitals are not equipped to support patients in a health crisis [34]. Specifically, depression and social anxiety can derail careers, hobbies, and social connections. In these challenging scenarios, adopting effective strategies play a vital role. Some studies suggests that government can facilitate people during the pandemic through following socio-economic policies: collaboration, response planning and community engagement, social and financial support, cash transfers, social services, generate opportunities, and development of online learning platforms [35]. Similarly, recent a study indicates that the way to more sustainable and resilient societies is revitalizing local industries, online food delivery, and online business operations [28]. Behavioral approaches and mental health strategies such as social assistance, relaxation, exercise, hobbies, awareness, and spirituality play fundamental role in refreshing mind [7]. 

In developing countries, strategic thinking and planning as well as defining priorities are critical in addressing crises due to poor hygiene, insufficient health, social, and economic support. Pakistan also faces severe challenges to beat COVID-19 because of bad governance system and weak financial position [3]. One of the most powerful tools for solving such a critical condition is the selection of suitable policy measures [36]. 

### Research Gap

In regional comparison, the experts believe that the present pandemic outbreak will hardly hit developing nations, especially South Asian economies [37]. Hence, this study tries to bridge the gap due to the following reasons:

(1) A plethora of studies available on the pandemic focused on upper–middle and high-income countries [16,38,39], while studies related to developing countries are not adequate; hence, this requires deep investigation [3,8].
(2) The urgent need is to examine the issue by a far more comprehensive approach, taking into account all relevant factors together rather than depending on one particular element (health preventive measures) for a holistic perspective of the problem.
(3) There is proper gap in exploring the context-based strategies because cultural and other factors, such as social and economic situation, political environment, and cultural system must be considered when comparing strategies across nations [40,41,42].
(4) There is lack of reliable framework in the pandemic literature that present and compile major containment strategies based on their significance level.

To fill these gaps, Interpretive Structural Modeling (ISM) is employed to address these critical issues [43].

## 3. Methodology

The current study approaches various steps to identify the significant strategies for containing pandemic. 

### 3.1. Exploring Socio-Economic and Health Strategies to Reduce Pandemic Stress

The purpose of the study is to propose key strategies for supporting socio-economic and health system in pandemic. The aim is achieved by investigating policies from the field of social psychology, sociology, health and policy, economic policy, and administration. Various bibliographic databases such as Google Scholar, Emerald, Wiley, Web of Science, Science Direct, EBSCO, Taylor and Francis, and Scopus were used to find the keywords. The inclusion and exclusion criteria are necessary for systematic literature studies [44]. The inclusion criteria consist of the following key points: (a) articles whose abstracts and titles match with our study keywords and papers published in high quality journals in English; (b) articles based on systematic literature focusing on pandemic containment policies in developing countries; and (c) studies that have been published in the last 5 years. In addition, the exclusion criteria are set on following grounds: (a) studies focusing mainly on quantitative data analysis rather than qualitative factors, (b) review articles and conference proceedings; and (c) published articles in languages other than English, and papers which were not matched with our study’s main objectives. The relevant articles were found from well-known databases such as Springer, Taylor and Francis, Wiley, and Scopus by searching the keywords: “social”, “economic”, “health”, “strategy”, “pandemic”, “socioeconomic crisis”, “health crisis”, and “pandemic containment strategies”. As result, a total of 14 strategies were identified, which are highlighted in Table 1.

### 3.2. Application of Interpretative Structural Modeling (ISM)

This approach has the ability to solve complex issues through a well-organized and logical way [101]. ISM method is basically and expert-based approach which utilizes various management techniques, such as brain storming, nominal technique, etc., to delineate sophisticated issues. The MCDM approaches assist decision makers in identifying, analyzing, and ranking alternatives based on a decision problem’s assessment of numerous criteria. To satisfy our study objectives, a variety of Multi-Criteria Decision Making (MCDM) approaches such as Analytic Hierarchy Process (AHP), Analytic Network Process (ANP), structural equation modeling (SEM), and DEMATEL are available. SEM is a set of mathematical techniques used to construct a theoretical framework, but it basically requires a large amount of data. The DEMATEL method illustrates the correlation among the causal-effect variables, but it does not provide hierarchy levels. Similarly, the AHP technique does not express inter-relationships among variables but is used to draw hierarchy level among factors. The ANP method has the ability to explain a causal relation between the variables, but the increased number of parameters gives greater inconsistency in the results. Previous studies used total interpretive structural modeling to assess the aspects that impact epidemiological features of pandemic [15]. Academic scholars frequently use modeling approaches for crisis evaluation and emergency management. Such modeling may generate prospective future scenarios based on current certain event conditions and potential uncertain event conditions, allowing network analysis to find appropriate controls for undesirable consequences [102]. Scenario modeling for emergency preparations [103], earthquake emergency management effectiveness [104], logistics planning during a flood emergency [105], and hurricane disaster emergency responses are a few examples of applications for scenario analysis in emergency management. Similar to these earlier emergency situations, the COVID-19 crisis could also be impacted by a number of complex factors or events, such as unpredictable objective factors beyond human control (such as the mutation of COVID-19 or the timing and location of the outbreak), as well as subjective factors brought on by serious risk prevention negligence (such as the delayed release of epidemic information and insufficient public education) [106].

These elements or occurrences are typically dynamic, urgent, and dependent on one another. Additionally, a COVID-19 epidemic occurs in an area with a high degree of dynamic openness, which makes COVID-19 more complicated and unpredictable than other emergency situations and increases the difficulty of decisions about pandemic prevention and management. To establish a structured approach for COVID-19 emergency management and control in this situation, interpretive structural modeling could be a crucial tool. Thus, interpretive analysis of the efficacy of COVID-19 prevention and control may give managers a multifaceted and thorough understanding of epidemic emergency management, comprising important scenario identification, factors logic links, factor levels, and so on. Based on the following advantages, this methodology has been employed:

- It assists in the presentation of a complicated system in a straightforward manner.
- It interprets the underlying object.
- It converts vague and poorly expressed visual models of systems into visible, clearer ones, which contribute to the construction of a theory.
- It makes the structure within a system easier and more understandable.

The ISM process can be classified into many steps:

- Step 1: Factor identification

The pandemic containment strategies are identified from the prior literature studies.

- Step 2: Creating contextual relationships

In this stage, the expert team was informed of the research objectives, and it helped them to construct the inter-relationships accurately among the identified strategies.

- Step 3: Establishing structural self-interaction matrix (SSIM)

The correlation matrix is constructed based on expert judgment after establishing a pairwise relationship among variables.

- Step 4: Developing a final reachability matrix and transitivity check

On the basis of the replies collected in Steps 2 and 3, an initial reachability matrix is created using the binary digits. Then, the final reachability matrix is constructed by using the transitivity rule.

- Step 5: Level partitions

The antecedent and reachable sets for each one of the 14 factors are derived through the final reachability matrix.

- Step 6: Development of diagraph

After placing the variables at their appropriate levels, a directed graph or diagraph is formed. According to the reachability matrix, direct linkages are established based on relationships.

- Step 7: ISM hierarchical model

**Figure 1:**
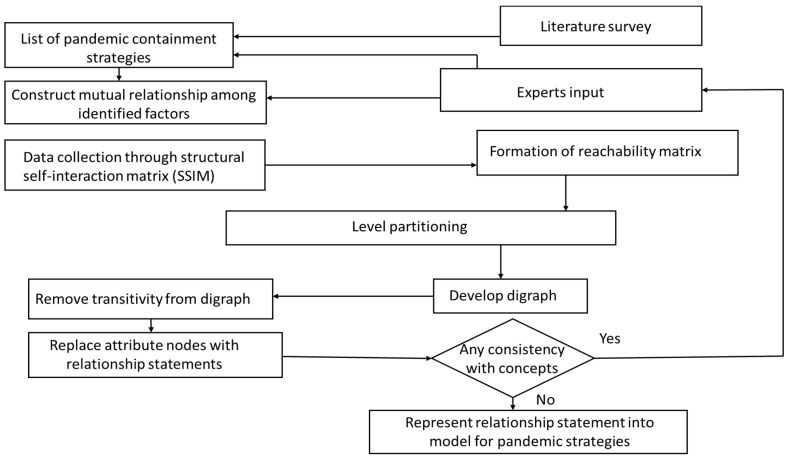
Step-by-step approach.

The outcome of level partitions helps to generate hierarchy model. The arrows pointing from i to j indicate a link between the factors (strategies) j and i. A digraph is the result of this process. Then, validity and conceptual consistency of the ISM model were checked. The above steps are shown in Figure 1.

### 3.3. SSIM Construction

In this step, contextual relationships were established among identified strategies. A team of experts, consisting of three medical specialists, four economists, three psychologists, four social workers, and two health policymakers was organized. The panel members were specialists in their fields, having more than 10 years of working experience. Four letters were employed to express the relationships between strategies (i and j), as shown in Table 2.

V Strategy (i) achieves strategy (j);
A Strategy (j) achieves strategy (i).
X Strategy (i) and (j) helps each other.
O Strategy (i) and (j) are unrelated.

#### 3.3.1. Initial Reachability Matrix Construction (IRM)

In this stage, the initial reachability matrix is constructed by transforming the SSIM into binary values. The substitutions of 0 and 1 were applied according to the following rules:

(a) If the cell containing (i,j) in the SSIM matrix is “V”, then the (i,j) cell in the reachability matrix should be filled with 1 and with 0 for the (j,i) cell.

(b) If the cell containing (i,j) in the SSIM matrix is “A”, then the (i,j) cell in the reachability matrix should be filled with 0 and with 1 for the (j,i) cell

(c) If the cell containing (i,j) in the SSIM matrix is “X”, then both entries (i,j) and (j,i) in the reachability matrix should be filled with 1.

(d) If the cell containing (i,j) in the SSIM matrix is “O”, then both entries (i,j) and (j,i) in the reachability matrix should be filled with 0.

By replacing the relevant binary numbers into the SSIM, the initial reachability matrix (IRM) is obtained, which is shown in Table 3. 

#### 3.3.2. Constructing Final Reachability Matrix (FRM)

By removing the transitivity from the IRM, the final reachability matrix is developed as shows in Table 4. The ISM methodology Step 4 helps to achieve final reachability matrix (FRM). 

#### 3.3.3. Level Partitions

Next, the FRM data were utilized to divide the strategies into different categories in order to better display their relative importance in hierarchical layers. Similarly, the reachability and antecedent set for each strategy is developed using the FRM. The factor and other sources that impact it constitute the reachability set. The antecedent set consists of the set of the elements’ components as well as other factors influencing that factor. In addition, reachability and antecedent sets were combined to create the intersection set. The factors (strategies) for which reachability and antecedent sets are identical were placed at the top level, i.e., “strong leadership and control” and “awareness on social media”. The factors which have an assigned level are eliminated from the process. Then, the process is repeated again until all variables have an assigned level. The top-level strategies play an essential role in crisis management during the pandemic. The government and policy makers should focus more on leadership and media constructive role in combating pandemic challenges. The results of level partition after seven iterations are presented in Appendix A (Table A1, Table A2, Table A3, Table A4, Table A5, Table A6 and Table A7).

### 3.4. Classification of Strategies (MICMAC Analysis)

Finally, this stage is used to analyze the driving and dependence power of each element (strategy). The FRM depicts the driving and dependence power of each factor as shown in Table 4. The identified strategies are classified into four quadrants depending on their respective drive and dependence values. 

**Autonomous cluster:** The quadrant factors have low driving and dependency force. In this study, no factors (strategies) pertain to this group. 

**Dependent cluster:** This cluster has weak driving power but high dependence. These factors are placed in a higher layer of the hierarchy model and are less important. There are five factors: promoting online purchase behaviors (S3), social distancing (S4), online education (S5), sealing the border (S13), and generating demand for domestic consumption (S14). 

**Linkage cluster:** Linkage cluster includes those strategies having strong driving power as well as dependence power but are unstable. These factors usually exist in the middle of the ISM hierarchy model and are factors such as prioritize key employees (S6), work from home (S11), and explore new market opportunities (S12).

**Independent cluster:** This quadrant’s factors have strong drive power and low dependency force. There are seven strategies in this cluster, such as smart lockdown (S1), travel limitations (S2), providing unemployment benefits from government (S7), testing, tracing, and isolation (S8), strong leadership and government control (S9), and awareness on social media (S10). These factors create a foundation for other factors because various strategies rely on it. Therefore, these elements need special care and priority focus.

## 4. Study Results

**Figure 2:**
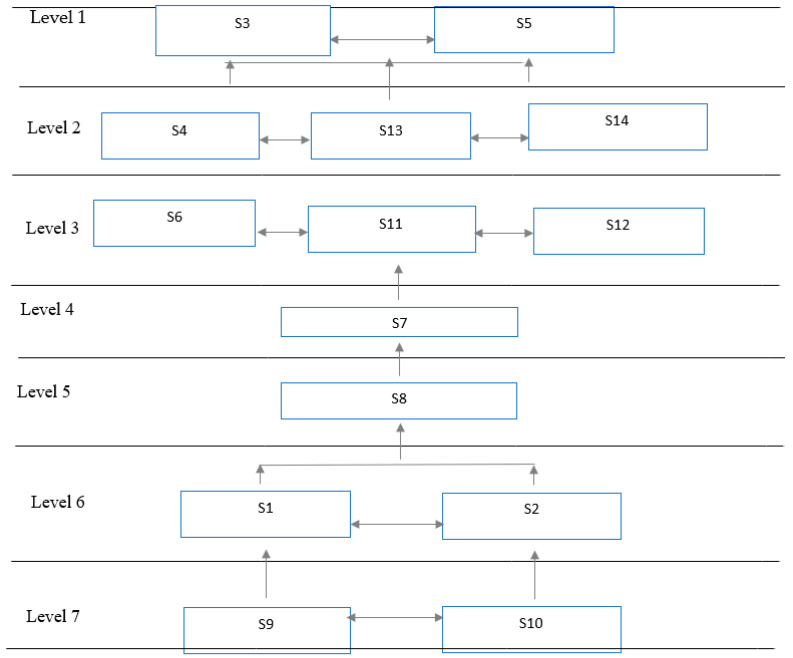
ISM model of key strategies for reducing socio–economic crisis during pandemic outbreak.

Developing countries face unprecedented challenges during the COVID-19 outbreak. To contain the virus spread, different countries implemented various non-pharmaceutical intervention. The main advantage of these strategies is that they protect against infections [38,107]. This study tries to identify key strategies for reducing socio-economic crises in resource-limited settings of Pakistan during the pandemic. The results of the ISM methodology extracted seven levels of pandemic strategies, as depicted in Figure 2. 

According to the ISM model, the strategies at the bottom level are more highly significant than others. 

Therefore, Level 7 strategies, i.e., “strong leadership and control” and “awareness on social media” play a pivotal role in the current pandemic crisis. The Pakistani government should give special focus and attention to these factors.

The reason lies in the fact that the public health system of any country is not fully autonomous from the government. In addition, good governance assists pandemic preparedness by continually investing in the healthcare system in order to minimize mortality, morbidity, and stress in a society [108,109,110,111]. Similarly, Sagan et al. [112] asserted that in Europe, effective governance has played a supportive role not only in health system but also in social and economic recovery. Therefore, the New Zealand Prime Minister, Jacinda Ardern, has been praised across the world for her swift response to COVID-19, which has enabled New Zealand to avoid the huge infections and fatalities that have affected many other nations [113]. The coronavirus pandemic may be the world’s greatest test of political leadership. Due to the lack of top leadership, India has experienced the worst COVID-19 crisis in the world as the adoption of containment strategies were inappropriate for the country’s scenario [45]. In addition, leadership in times of crisis prioritizes resources and responds through better communication skills [76,114]. 

Accordingly, at Level 6 “travel limitations” and “smart lockdown” strategies are considered the best approaches in containing virus, as developing countries cannot afford a strict lockdown strategy. Some advanced countries, such as the UK and Italy, have imposed strict lockdowns from July 11, 2020, to prevent the spread of virus. Their governments also stressed that people cancel unnecessary travel in the pandemic peak time [115]. The previous studies confirmed that such strategies are assisting governments throughout the world in limiting COVID-19 exposure [116,117]. The response of the Pakistani leadership to the pandemic has been remarkable because the strategies largely support the country’s environment, such as smart lockdown strategy [33], providing accurate information and an awareness campaign, online home delivery services, financial assistance, and support of businesses [2]. However, there is no ‘one size fits all’ policy that delivers desired results in every situation. For instance, the Indian Government imposed draconian lockdown in March 2020 that did not control the virus but unleashed a dramatic financial disaster and humanitarian catastrophe in the country. Therefore, India’s second wave pandemic crisis dealt a significant blow to prime minister Narendra Modi [45]. It is critical to take preventive measure to control epidemic without harming economy. Good governance advocates smart lockdown strategy in weak economies to restore the production processes [118]. 

Level 6 leads to Level 5 strategy “testing, tracing, and isolation”. This is considered an effective preventive technique because the country has high importation cases that need early diagnosis of the infected people to prevent spreading, as well as surveillance steps to deter further virus transmission. This technique is widely adopted in China, Singapore, Japan, and Thailand [17,119]. Notably, this strategy is crucial for developing the nation’s fragile health system [120,121]. For instance, the Pakistani health infrastructure and medical staff are vulnerable to the current pandemic. Therefore, the government of Pakistan should implement this strategy to avoid excessive burden on healthcare system. 

At Level 4, this study found “unemployment benefits from government” to be an important strategy to relieve the economic unrest, which is more appealing in developing economies. Even so, COVID-19 has caused millions of Americans to become jobless, i.e., (wage earners to part-time workers) with an estimated range between 13 and 36 million people [122]. As a result, a multi-dimensional disaster has emerged from this pandemic in the form of unemployment, paralyzed healthcare system, and less personal and community support [122]. Hence, people will require security in order to remain safe and healthy [123], which comes in the form of food, social benefits, and other socio-economic support. In this vein, the UK government introduced a number of schemes—job retention scheme (JRS) and self-employment income support scheme (SEISS)—to support the economy, workers, and businesses [65]. The government of Pakistan already allocated a financial package of PKR 2.1 trillion in March 2020, especially for low-income families, jobless workers, and economic fallout from the pandemic outbreak. However, the government still needs more packages to reach the vulnerable people in this ongoing global crisis. 

At Level 3, our study revealed three important strategies: prioritize key employees, work from home, and explore new market opportunities, which are beneficial during a pandemic crisis. As a previous study identified, 37 % of virus transmission occurred in firms and education sectors [124]. Although people of all ages are vulnerable to COVID-19, older people are at a higher risk of contracting drastic illness as a result of physiological conditions associated with aging and potential underlying physical conditions [125]. Therefore, state departments in many regions have issued a notice to workplaces to take special care of aged workforce’. Younger people have a stronger immune system, making them less susceptible to the virus. One of the bright side of the pandemic is that it has opened up the door for new business opportunities as consumers adapt to post-COVID life. For instance, textile companies can make personal protective equipment’s (PPE) and respirators. Several textile sectors have already produced respirators and other medical equipment in China. In addition, perfume companies and distilleries have opportunities to manufacture hand sanitizers [126]. The current outbreak has also changed the occupational status such as working from home; thus, many nations have adopted this strategy to eliminate human contact [87]. Level 3 factors lead to Level 2 strategies: social distancing, sealing the border, and generating demand for domestic consumption. The state governments in many nations have divided cities into various geographical regions based on the severity of the Coronavirus spread. This is called the ‘traffic light’ model. Similarly, Mexico has implemented a ‘traffic light’ system based on a risk assessment, while, China has sealed its borders to prevent the spread of COVID-19 by imposing travel restrictions on foreign visitors [127]. The increase in social connections prompted by enormous transportation and gathering results in rapid transmission of the virus [128,129]. Thus, the best strategy to contain the virus transmission is adopting social distancing measures or regulations as well as closing or reducing mobility in transportation networks where the transmission rate is higher [129,130]. The border closure in the pandemic has interrupted consumer market dynamics. Therefore, the government should incentivize the businesses to optimize the supply chain costs [131]. Border closure and other containment strategies have confined consumers to access international markets. Thus, the paradigm of domestic consumption has geared up. Firms should make a policy to produce goods to meet domestic demand. 

Finally, Level 2 strategies lead to Level 1 such as “promoting online purchase behavior” and “online education”. The previous studies identified that sealing the borders and social distancing have encouraged online shopping [132]. As customers are considered key shareholders in any business, decline in demand will have direct impact in a firm’s profitability, so they should be encouraged to make purchases through the internet. However, online shopping in developing countries such as Pakistan is still in the nascent stage of adoption, so the government should support online businesses for economic recovery. Similarly, social distancing, lockdown, and other virus containment strategies have changed the structure of education. For example, nurseries, childcare centers, preschools, universities, and colleges in Norway, US, UK, and other countries have shifted their traditional way of learning to digital learning and teaching [133]. 

## 5. Discussion

The COVID-19 pandemic has negative socio-economic and health implications. Many countries are experimenting with a variety of traditional approaches and strategies to combat the disease. The most prominent policies are social distancing, face masks [134], and lockdown [135]. These measures are helpful in preventing the spread of SARS-CoV-2, but they also have adverse implications in daily life. Previous outbreaks, for example, have underlined the need for policymakers to keep individual avoidance activities to a minimum, so this might impose significant economic costs while not necessarily limiting virus transmission [136]. There is an urgent need for broad strategies at the regional and context levels that offer public health preventive measures at the primary level as well as a supportive role in other parameters (e.g., socio-cultural and economic, institutional, media, and improved societies) [38]. Although several authors have investigated various strategies to curb the pandemic, there is paucity of studies from developing countries context. In addition, the successful implementation of policy interventions require a suitable framework, which is missing in the emerging literature. Therefore, this study investigated and prioritized 14 major strategies with the help of the ISM approach. Using the ISM technique, the study formulated seven layers of strategies through a hierarchy model.

The results indicate that “top leadership” and “awareness on social media” have a high driving power as compared with other factors in limiting pandemic challenges, which depicted the real scenario of developing countries such as Pakistan. Because good governance can contribute to all segments of society, i.e., education, economy, health, social welfare, media coverage, etc., the current outbreak provides an opportunity for leaders to adopt a set of best practices, systems, and policies in overcoming difficulties, anxieties, and to manage the crisis. The strategies of Pakistan’s top leadership in times of pandemic crisis is very impressive compared with its neighboring nations; therefore, the country has been placed as the third world’s best country in terms of efficient outbreak control [137]. However, to pave the way for an economic recovery and stability in all sectors, the government still needs more innovative and skillful approaches. 

## 6. Managerial Implications

This study is valuable to Pakistani government, policymakers, and practitioners. The results suggests that “strong leadership” and “awareness on social media” have larger impact in resolving the socio-economic and health issues in pandemic. The lessons learnt from the study advocate that good governance delivers outstanding performance in hard times. The study implies that crisis management requires transparency and clear and true information. For instance, Singapore and New Zealand have turned into an international role models for dealing the pandemic. Therefore, effective governance system can build a resilient approach to tackle crises such as hunger, poverty, unemployment, etc. [138,139]. The hierarchy levels of pandemic strategies also assist policymakers in prioritizing and implementing the key strategies in a timely manner. This way, it can support the creation of a visible roadmap by formulating major policies. Furthermore, macro- and micro-economic development strategies will be critical in curbing socio-economic challenges [140]. 

### 6.1. Practical Implications

Governments of many nations have introduced stringent standards and procedures to overcome their economic loss as the epidemic has spread internationally, and an economic lockdown is in place to protect people’s mental health. The two sectors that contribute most to the expansion of the national economy are manufacturing and construction. The government should create regulations for these sectors. Employees should maintain social distance from one another at work. After work hours, the tools should be cleaned well, and employees who are not necessary should work from home. Additionally, entry and exit locations should have disinfection walk-through gates installed. Tools and equipment used in the production of goods must be cleaned and sanitized. Through the adoption of these protocol measures, the economic situation of the country could be restored, and social well-being could be achieved. Also, implementing these socio-economic and health policies can create balance in every sector of a country.

### 6.2. Theoretical Contribution

This study is one of the known attempts that considers all context-based strategies to reduce the country’s social, economic, and health issues in the pandemic period. In the emerging pandemic literature, many studies emphasized only public health policies [16,141], while others integrated only social and health policies [142] or social and economic measures [143]. However, less attention has been paid to integrating socio-economic and health strategies. As health authorities have realized, viral diseases not only cause public health system strain but also create long-term psychological stress and anxiety [144]. In this uncertain situation, learning from other countries’ experiences and doing things differently might benefit nations, and indigenous solutions are required for maintaining balance and stability in COVID-19. Through analysis of the pandemic scenario, this study investigated suitable strategies that could facilitate all segments of life. There are many studies that employed the ISM–MICMAC technique in the manufacturing sector [145], service sector [146], tourism industry [147], and construction sector [148], while the applicability of the ISM approach in emerging pandemic studies is limited, which needs further exploration [43]. It is one of the prime studies that identify broad strategies for reducing pandemic issues through hierarchy levels on the basis of their significance level.

## 7. Conclusions

The pandemic has wreaked chaos in human life on a level never before seen. It has revealed cracks in almost every sector causing unprecedented level of health and economic loss. The impact of this viral disease is disproportionately unequal depending on social and economic factors. The developing countries are more exposed to the crisis due to less socioeconomic and health support. The urgent need is to identify diverse policy measures to create a more coordinated balance approach that can halt the spread of virus. Therefore, this study identifies a set of policies which can give support in current pandemic period. In this study, we identified 14 strategies after conducting a thorough literature survey and heeding experts’ suggestions. The study results represent seven levels in the hierarchy model. The final results suggests that top leadership and social media have critical roles in pandemic crisis in developing countries such as Pakistan. However, promoting online purchase behavior and online education are the least important strategies in reducing pandemic issues. 

### 7.1. Limitations

Although this study provides significant implications, it has also several drawbacks. First, this study considered very limited factors, which might not capture all the parameters of the socio-economic and health system in pandemic period. Second, this study cannot be generalized to other nations due to the cultural system, social protection mechanism, and other dimensions [39]. Further, this study employed the ISM approach which is not statistically validated and verified. Finally, the ISM method presents relationships among factors through binary values (0, 1), which are not specified in terms of weak, strong, or low relationships. 

### 7.2. Future Research Scope

Future studies should generate a comprehensive list of factors that can give tremendous output. Thus, future research work can apply other rigorous techniques such as structural equation modeling (SEM) for testing the results. Therefore, combined approaches, such as fuzzy logic and ISM are suggested.

## Figures and Tables

**Table 1:** List of identified strategies to counter pandemic crisis.

S. NO	Strategies	References	Description
**S1**	Smart lockdown	[38,45]	The smart lockdown is a policy to enforce complete lockdown in selected areas where the risk index is high compared with other localities.
**S2**	Travel limitations	[46,47,48,49]	Unnecessary travel limitation is a good option to control the pandemic.
**S3**	Promoting online purchase behaviors	[50,51,52]	To control the spread of virus, online purchase behavior should be promoted.
**S4**	Social distancing	[52,53,54,55,56,57,58]	Social distancing is reducing physical interaction between people, and it lowers the chances of spreading it between people.
**S5**	Online education	[59,60,61]	This startegy is also helpful in curbing virus transmission.
**S6**	Prioritize key employees	[52]	According to this approach, organizations should reduce staff to minimize the risk of infection in the workforce.
**S7**	Provide unemployment benefits from the government	[62,63,64,65,66]	The government should provide incentive schemes to those who lost their jobs in the pandemic.
**S8**	Testing, tracing, and isolation	[67,68,69,70,71]	This technique is very valuable in early diagnosis of infected people.
**S9**	Strong leadership and government control	[72,73,74,75,76,77,78]	Committed government agenda can provide solutions in times of crisis such as the current pandemic.
**S10**	Awareness on social media	[79,80,81,82]	Companign on social media about pandemic containment policies can educate people how to keep healthy and safe.
**S11**	Work from home	[52,83,84,85,86,87]	This strategy can reduce workers’ exposure to the COVID-19 virus.
**S12**	Explore new market opportunities	[88,89,90,91,92]	COVID-19 has opened new market opportunities, such as online food delivery services, online tutoring, digital marketing, etc.
**S13**	Sealing the border	[93,94,95,96]	Governments has successfully sealed off their countries in the past to prevent disease.
**S14**	Generating demand for domestic consumption	[97,98,99,100]	Because of the border closure, the production and consumption of domestic goods has raised, which is a positive sign for local firms.

**Table 2:** Structural Self-interaction Matrix (SSIM).

No.	Variables	1	2	3	4	5	6	7	8	9	10	11	12	13	14
**S1**	Smart lockdown		X	V	V	V	O	V	V	A	A	V	O	V	V
**S2**	Travel limitations			V	V	O	V	V	O	A	A	V	V	V	V
**S3**	Promoting online purchase behaviors				A	V	A	A	O	A	A	A	A	A	A
**S4**	Social distancing					V	A	A	A	A	O	A	A	X	V
**S5**	Online education						O	A	A	A	A	A	A	A	O
**S6**	Prioritize key employees							A	A	A	A	X	A	V	A
**S7**	Provide unemployment benefits from government								A	A	O	O	V	V	V
**S8**	Testing, tracing, and isolation									A	A	O	O	O	V
**S9**	Strong leadership and government control										X	V	O	V	V
**S10**	Awareness on social media											V	V	V	O
**S11**	Work from home												X	V	V
**S12**	Explore new market opportunities													V	V
**S13**	Sealing the border														A
**S14**	Generating demand for domestic consumption														

X: In SSIM Table 2, Strategy 1 and Driver 2 help achieve each other, which means that “Smart lockdown” and “travel limitation” help achieve each other; therefore, this pairwise relationship is given as “X” symbol. V: Strategy 2 (S2) helps to influence Strategy 3 (S3), which means that as “travel limitation increases, the “online purchase behaviors” increases as well. Thus, this type of relationship is denoted as “V”. A: Strategy 4 (S4) helps achieve Strategy 3 (S3), which means that “social distancing” affects “promoting online purchase behavior”. Thus, such relationships are known as “A”. O: Strategy 5 (S5) and Strategy 6 (S6) have no relationship, which means “online education and “prioritize key employees” are unrelated, so this relationships is given symbol “O”.

**Table 3:** Initial reachability matrix (IRM).

S.#	1	2	3	4	5	6	7	8	9	10	11	12	13	14
1	1	1	1	1	1	0	1	1	0	0	1	0	1	1
2	1	1	1	1	0	1	1	0	0	0	1	1	1	1
3	0	0	1	0	1	0	0	0	0	0	0	0	0	0
4	0	0	1	1	1	0	0	0	0	0	0	0	1	1
5	0	0	0	0	1	0	0	0	0	0	0	0	0	0
6	0	0	1	1	0	1	0	0	0	0	1	0	1	1
7	0	0	1	1	1	1	1	0	0	0	0	1	1	1
8	0	0	0	1	1	1	1	1	0	0	1	1	0	1
9	1	1	1	1	1	1	1	1	1	1	1	0	1	1
10	1	1	1	0	1	1	0	1	1	1	1	1	1	0
11	0	0	1	1	1	1	0	0	0	0	1	1	1	1
12	0	0	1	1	1	1	0	0	0	0	1	1	1	1
13	0	0	1	1	1	0	0	0	0	0	0	0	1	0
14	0	0	1	0	0	0	0	0	0	0	0	0	1	1

**Table 4:** Final reachability matrix (FRM).

S. NO	1	2	3	4	5	6	7	8	9	10	11	12	13	14	DRIVING POWER
1	1	1	1	1	1	* 1	1	1	0	0	1	* 1	1	1	12
2	1	1	1	1	* 1	1	1	* 1	0	0	1	1	1	1	12
3	0	0	1	0	1	0	0	0	0	0	0	0	0	0	2
4	0	0	1	1	1	0	0	0	0	0	0	0	1	1	5
5	0	0	* 1	0	1	0	0	0	0	0	0	0	0	0	2
6	0	0	1	1	* 1	1	0	0	0	0	1	* 1	1	1	8
7	0	0	1	1	1	1	1	0	0	0	* 1	1	1	1	9
8	0	0	* 1	1	1	1	1	1	0	0	1	1	* 1	1	10
9	1	1	1	1	1	1	1	1	1	1	1	* 1	1	1	14
10	1	1	1	* 1	1	1	* 1	1	1	1	1	1	1	* 1	14
11	0	0	1	1	1	1	0	0	0	0	1	1	1	1	8
12	0	0	1	1	1	1	0	0	0	0	1	1	1	1	8
13	0	0	1	1	1	0	0	0	0	0	0	0	1	* 1	5
14	0	0	1	* 1	* 1	0	0	0	0	0	0	0	1	1	5
DEPENDENCE POWER	4	4	14	12	14	9	6	5	2	2	9	9	12	12	

* indicates transitivity after analysis.

## Data Availability

Associated data are provided in the manuscript.

## Appendix A

**Table A1:** Level partitions (Iteration I).

S. No.	Reachability Set	Antecedent Set	Intersection set	Level
S1	1,2,3,4,5,6,7,8,11,12,13,14	1,2,9,10	1,2	
S2	1,2,3,4,5,6,7,8,11,12,13,14	1,2,9,10	1,2	
S3	3,5	1,2,3,4,5,6,7,8,9,10,11,12,13,14	3,5	I
S4	3,4,5,13,14	1,2,4,6,7,8,9,10,11,12,13,14	4,13,14	
S5	3,5	1,2,3,4,5,6,7,8,9,10,11,12,13,14	3,5	I
S6	3,4,5,6,11,12,13,14	1,2,6,7,8,9,10,11,12	6,11,12	
S7	3,4,5,6,7,11,12,13,14	1,2,7,8,9,10	7	
S8	3,4,5,6,7,8,11,12,13,14	1,2,8,9,10	8	
S9	1,2,3,4,5,6,7,8,9,10,11,12,13,14	9,10	9,10	
S10	1,2,3,4,5,6,7,8,9,10,11,12,13,14	9,10	9,10	
S11	3,4,5,6,11,12,13,14	1,2,6,7,8,9,10,11,12	6,11,12	
S12	3,4,5,6,11,12,13,14	1,2,6,7,8,9,10,11,12	6,11,12	
S13	3,4,5,13,14	1,2,4,6,7,8,9,10,11,12,13,14	4,13,14	
S14	3,4,5,13,14	1,2,4,6,7,8,9,10,11,12,14	4,13,14	

**Table A2:** Level partitions (Iteration II).

S. No.	Reachability Set	Antecedent Set	Intersection set	Level
S1	1,2,4,6,7,8,11,12,13,14	1,2,9,10	1,2	
S2	1,2,4,6,7,8,11,12,13,14	1,2,9,10	1,2	
S4	4,13,14	1,2,4,6,7,8,9,10,11,12,13,14	4,13,14	II
S6	4,6,11,12,13,14	1,2,6,7,8,9,10,11,12	6,11,12	
S7	4,6,7,11,12,13,14	1,2,7,8,9,10	7	
S8	4,6,7,8,11,12,13,14	1,2,8,9,10	8	
S9	1,2,4,6,7,8,9,10,11,12,13,14	9,10	9,10	
S10	1,2,4,6,7,8,9,10,11,12,13,14	9,10	9,10	
S11	4,6,11,12,13,14	1,2,6,7,8,9,10,11,12	6,11,12	
S12	4,6,11,12,13,14	1,2,6,7,8,9,10,11,12	6,11,12	
S13	4,13,14	1,2,4,6,7,8,9,10,11,12,13,14	4,13,14	II
S14	4,13,14	1,2,4,6,7,8,9,10,11,12,14	4,13,14	II

**Table A3:** Level partitions (Iteration III).

S. No.	Reachability Set	Antecedent Set	Intersection set	Level
S1	1,2,6,7,8,11,12	1,2,9,10	1,2	
S2	1,2,6,7,8,11,12	1,2,9,10	1,2	
S6	6,11,12	1,2,6,7,8,9,10,11,12	6,11,12	III
S7	6,7,11,12	1,2,7,8,9,10	7	
S8	6,7,8,11,12	1,2,8,9,10	8	
S9	1,2,6,7,8,9,10,11,12	9,10	9,10	
S10	1,2,7,8,9,10,11,12	9,10	9,10	
S11	6,11,12	1,2,6,7,8,9,10,11,12	6,11,12	III
S12	6,11,12	1,2,6,7,8,9,10,11,12	6,11,12	III

**Table A4:** Level partitions (Iteration IV).

S. No.	Reachability Set	Antecedent Set	Intersection Set	Level
S1	1,2,7,8	1,2,9,10	1,2	
S2	1,2,7,8	1,2,9,10	1,2	
S7	7	1,2,7,8,9,10	7	IV
S8	7,8	1,2,8,9,10	8	
S9	1,2,7,8,9,10	9,10	9,10	
S10	1,2,7,8,9,10	9,10	9,10	

**Table A5:** Level partitions (Iteration V).

S. No.	Reachability Set	Antecedent Set	Intersection set	Level
S1	1,2,8	1,2,9,10	1,2	
S2	1,2,8	1,2,9,10	1,2	
S8	8	1,2,8,9,10	8	V
S9	1,2,8,9,10	9,10	9,10	
S10	1,2,8,9,10	9,10	9,10	

**Table A6:** Level partitions (Iteration VI).

S. No.	Reachability Set	Antecedent Set	Intersection Set	Level
S1	1,2	1,2,9,10	1,2	VI
S2	1,2	1,2,9,10	1,2	VI
S9	1,2,9,10	9,10	9,10	
S10	1,2,9,10	9,10	9,10	

**Table A7:** Level partitions (Iteration VII).

S. No.	Reachability Set	Antecedent Set	Intersection Set	Level
S9	9,10	9,10	9,10	VII
S10	9,10	9,10	9,10	VII

## References

[1] Xu Z., Shi L., Wang Y., Zhang L., Huang L., Zhang C. (2020). Pathological finding of COVID–19 associated with acute respiratory distress syndrome. Lancet Respir. Med..

[2] Shafi M., Liu J., Ren W. (2020). Impact of COVID–19 pandemic on micro, small, and medium–sized Enterprises operating in Pakistan. Res. Glob..

[3] Rasheed R., Rizwan A., Javed H., Sharif F., Zaidi A. (2021). Socio–economic and environmental impacts of COVID–19 pandemic in Pakistan—An integrated analysis. Environ. Sci. Pollut. Res..

[4] Van Bavel J.J., Baicker K., Boggio P.S., Capraro V., Cichocka A., Cikara M., Crockett M.J., Crum A.J., Douglas K.M., Druckman J.N. (2020). Using social and behavioural science to support COVID–19 pandemic response. Nat. Hum. Behav..

[5] Dryhurst S., Schneider C.R., Kerr J., Freeman A.L., Recchia G., Van Der Bles A.M., Spiegelhalter D., Van Der Linden S. (2020). Risk perceptions of COVID–19 around the world. J. Risk Res..

[6] Richter I., Avillanosa A., Cheung V., Goh H.C., Johari S., Kay S., Maharja C., Nguyễn T.H., Pahl S., Sugardjito J. (2021). Looking through the COVID–19 window of opportunity: Future scenarios arising from the COVID–19 pandemic across five case study sites. Front. Psychol..

[7] Garcini L.M., Rosenfeld J., Kneese G., Bondurant R.G., Kanzler K.E. (2022). Dealing with distress from the COVID-19 pandemic: Mental health stressors and coping strategies in vulnerable latinx communities. Health Soc. Care Community.

[8] Chakraborty I., Maity P. (2020). COVID–19 outbreak: Migration, effects on society, global environment and prevention. Sci. Total Environ..

[9] Collivignarelli M.C., Abbà A., Bertanza G., Pedrazzani R., Ricciardi P., Miino M.C. (2020). Lockdown for CoViD–2019 in Milan: What are the effects on air quality?. Sci. Total Environ..

[10] Messner W. (2020). The institutional and cultural context of cross–national variation in COVID–19 outbreaks. Medrxiv.

[11] Ayebare R.R., Flick R., Okware S., Bodo B., Lamorde M. (2020). Adoption of COVID–19 triage strategies for low–income settings. Lancet Respir. Med..

[12] Hopman J., Allegranzi B., Mehtar S. (2020). Managing COVID–19 in low–and middle–income countries. Jama.

[13] Shuchman M. (2020). Low–and middle–income countries face up to COVID–19. Nat. Med..

[14] Leidecker J.K., Bruno A.V. (1984). Identifying and using critical success factors. Long Range Plan..

[15] Lakshmi Priyadarsini S., Suresh M. (2020). Factors influencing the epidemiological characteristics of pandemic COVID 19: A TISM approach. Int. J. Healthc. Manag..

[16] Pozo–Martin F., Weishaar H., Cristea F., Hanefeld J., Bahr T., Schaade L., El Bcheraoui C. (2021). The impact of non–pharmaceutical interventions on COVID–19 epidemic growth in the 37 OECD member states. Eur. J. Epidemiol..

[17] Chen H., Shi L., Zhang Y., Wang X., Sun G. (2021). A cross–country core strategy comparison in China, Japan, Singapore and South Korea during the early COVID–19 pandemic. Glob. Health.

[18] Alanezi F., Aljahdali A., Alyousef S.M., Alrashed H., Mushcab H., AlThani B., Alghamedy F., Alotaibi H., Saadah A., Alanzi T. (2020). A comparative study on the strategies adopted by the United Kingdom, India, China, Italy, and Saudi Arabia to contain the spread of the COVID–19 pandemic. J. Healthc. Leadersh..

[19] Bourdin S., Ben Miled S., Salhi J. (2022). The drivers of policies to limit the spread of COVID–19 in Europe. J. Risk Financ. Manag..

[20] Islam S.N., Cheng H.W.J., Helgason K., Hunt N., Kawamura H., LaFleur M. (2020). Variations in COVID Strategies: Determinants and Lessons.

[21] Hsiang S., Allen D., Annan–Phan S., Bell K., Bolliger I., Chong T., Druckenmiller H., Huang L.Y., Hultgren A., Krasovich E. (2020). The effect of large–scale anti–contagion policies on the COVID–19 pandemic. Nature.

[22] Haug N., Geyrhofer L., Londei A., Dervic E., Desvars–Larrive A., Loreto V., Pinior B., Thurner S., Klimek P. (2020). Ranking the effectiveness of worldwide COVID–19 government interventions. Nat. Hum. Behav..

[23] Baveja A., Kapoor A., Melamed B. (2020). Stopping Covid–19: A pandemic–management service value chain approach. Ann. Oper. Res..

[24] Berger Z.D., Evans N.G., Phelan A.L., Silverman R.D. (2020). Covid–19: Control measures must be equitable and inclusive. BMJ.

[25] García–Álvarez L., de la Fuente–Tomás L., García–Portilla M.P., Sáiz P.A., Lacasa C.M., Dal Santo F., González–Blanco L., Bobes–Bascarán M.T., García M.V., Vázquez C.Á. (2020). Early psychological impact of the 2019 coronavirus disease (COVID–19) pandemic and lockdown in a large Spanish sample. J. Glob. Health.

[26] Rossi R., Socci V., Talevi D., Mensi S., Niolu C., Pacitti F., Di Marco A., Rossi A., Siracusano A., Di Lorenzo G. (2020). COVID–19 pandemic and lockdown measures impact on mental health among the general population in Italy. Front. Psychiatry.

[27] Gulati G., Kelly B.D. (2020). Domestic violence against women and the COVID–19 pandemic: What is the role of psychiatry?. Int. J. Law Psychiatry.

[28] Rasul G., Nepal A.K., Hussain A., Maharjan A., Joshi S., Lama A., Gurung P., Ahmad F., Mishra A., Sharma E. (2021). Socio–Economic Implications Of Covid–19 Pandemic In South Asia: Emerging Risks And Growing Challenges. Front. Sociol..

[29] Thakur V. (2022). Locating temporary waste treatment facilities in the cities to handle the explosive growth of HCWs during pandemics: A novel Grey–AHP–OCRA hybrid approach. Sustain. Cities Soc..

[30] Thakur V. (2021). Framework for PESTEL dimensions of sustainable healthcare waste management: Learnings from COVID–19 outbreak. J. Clean. Prod..

[31] Hossain M.K., Thakur V., Kazancoglu Y. (2022). Developing a resilient healthcare supply chain to prevent disruption in the wake of emergency health crisis. Int. J. Emerg. Mark..

[32] Hossain M.K., Thakur V., Mangla S.K. (2021). Modeling the emergency health–care supply chains: Responding to the COVID–19 pandemic. J. Bus. Ind. Mark..

[33] Wang C., Wang D., Abbas J., Duan K., Mubeen R. (2021). Global financial crisis, smart lockdown strategies, and the COVID–19 spillover impacts: A global perspective implications from Southeast Asia. Front. Psychiatry.

[34] Saraceno B., van Ommeren M., Batniji R., Cohen A., Gureje O., Mahoney J., Sridhar D., Underhill C. (2007). Barriers to improvement of mental health services in low–income and middle–income countries. Lancet.

[35] Ozili P. (2020). COVID–19 in Africa: Socio–economic impact, policy response and opportunities. Int. J. Sociol. Soc. Policy.

[36] Sohil F., Sohail M.U., Shabbir J. (2021). COVID–19 in Pakistan: Challenges and priorities. Cogent Med..

[37] Islam M., Jannat A., Al Rafi D.A., Aruga K. (2020). Potential Economic Impacts of the COVID–19 Pandemic on South Asian Economies: A Review. World.

[38] Regmi K., Lwin C.M. (2021). Factors Associated with the Implementation of Non–Pharmaceutical Interventions for Reducing Coronavirus Disease 2019 (COVID–19): A Systematic Review. Int. J. Environ. Res. Public Health.

[39] Keogh–Brown M.R., Jensen H.T., Edmunds W.J., Smith R.D. (2020). The impact of Covid–19, associated behaviours and policies on the UK economy: A computable general equilibrium model. SSM–Popul. Health.

[40] Miles D., Stedman M., Heald A. (2020). Living with COVID–19: Balancing costs against benefits in the face of the virus. Natl. Inst. Econ. Rev..

[41] Desierto D., Koyama M. (2020). Health vs. Economy: Politically Optimal Pandemic Policy. J. Politi-Institutions Politi-Econ..

[42] Nyarko R., Boateng E., Kahwa I., Boateng P., Asare B. (2020). The impact on public health and economy using lockdown as a tool against COVID–19 pandemic in Africa: A perspective. J. Epidemiol. Public Health Rev..

[43] Basit A., Khan Niazi A.A., Fiaz Qazi T., Rao Z.-u.-R., Shaukat M.Z. (2021). Structural Modeling on the Determinants of Effectiveness of SOPs Containing COVID–19 in Mass Gatherings. Front. Psychol..

[44] Dresch A., Lacerda D.P., Antunes J.A.V. (2015). Design Science Research. Design Science Research.

[45] Ghosh J. (2020). A critique of the Indian government’s response to the COVID–19 pandemic. J. Ind. Bus. Econ..

[46] Gössling S., Scott D., Hall C.M. (2020). Pandemics, tourism and global change: A rapid assessment of COVID–19. J. Sustain. Tour..

[47] Kraemer M.U., Yang C.-H., Gutierrez B., Wu C.–H., Klein B., Pigott D.M., du Plessis L., Faria N.R., Li R., Open COVID-19 Data Working Group (2020). The effect of human mobility and control measures on the COVID–19 epidemic in China. Science.

[48] Hao F., Xiao Q., Chon K. (2020). COVID–19 and China’s hotel industry: Impacts, a disaster management framework, and post–pandemic agenda. Int. J. Hosp. Manag..

[49] Smith M.K.S., Smit I.P., Swemmer L.K., Mokhatla M.M., Freitag S., Roux D.J., Dziba L. (2021). Sustainability of protected areas: Vulnerabilities and opportunities as revealed by COVID–19 in a national park management agency. Biol. Conserv..

[50] Adolph C., Amano K., Bang–Jensen B., Fullman N., Wilkerson J. (2021). Pandemic politics: Timing state–level social distancing responses to COVID–19. J. Health Politics Policy Law.

[51] Alaimo L.S., Fiore M., Galati A. (2021). Measuring consumers’ level of satisfaction for online food shopping during COVID–19 in Italy using POSETs. Socio–Econ. Plan. Sci..

[52] Kashyap A., Raghuvanshi J. (2020). A preliminary study on exploring the critical success factors for developing COVID–19 preventive strategy with an economy centric approach. Manag. Res. J. Iberoam. Acad. Manag..

[53] Guo Y., Qin W., Wang Z., Yang F. (2021). Factors influencing social distancing to prevent the community spread of COVID–19 among Chinese adults. Prev. Med..

[54] Niu Y., Xu F. (2020). Deciphering the power of isolation in controlling COVID–19 outbreaks. Lancet Glob. Health.

[55] Triberti S., Durosini I., Pravettoni G. (2021). Social distancing is the right thing to do: Dark Triad behavioral correlates in the COVID–19 quarantine. Personal. Individ. Differ..

[56] Kretzschmar M.E., Rozhnova G., van Boven M. (2021). Isolation and contact tracing can tip the scale to containment of COVID–19 in populations with social distancing. Front. Phys..

[57] Yasir K.A., Liu W.-M. (2021). Social distancing mediated generalized model to predict epidemic spread of COVID–19. Nonlinear Dyn..

[58] Lee S.M., Lee D. (2020). Lessons learned from battling COVID–19: The Korean experience. Int. J. Environ. Res. Public Health.

[59] Chakraborty P., Mittal P., Gupta M.S., Yadav S., Arora A. (2021). Opinion of students on online education during the COVID-19 pandemic. Hum. Behav. Emerg. Technol..

[60] Mishra L., Gupta T., Shree A. (2020). Online teaching–learning in higher education during lockdown period of COVID–19 pandemic. Int. J. Educ. Res. Open.

[61] Jain D., Chakraborty P., Chakraverty S. (2018). Smartphone apps for teaching engineering courses: Experience and scope. J. Educ. Technol. Syst..

[62] Bienvenido–Huertas D. (2021). Do unemployment benefits and economic aids to pay electricity bills remove the energy poverty risk of Spanish family units during lockdown? A study of COVID–19–induced lockdown. Energy Policy.

[63] Larrimore J., Burkhauser R.V., Armour P. (2015). Accounting for income changes over the Great Recession relative to previous recessions: The impact of taxes and transfers. Natl. Tax J..

[64] Salgado M.F., Figari F., Sutherland H., Tumino A. (2014). Welfare compensation for unemployment in the Great Recession. Rev. Income Wealth.

[65] Brewer M., Tasseva I.V. (2021). Did the UK policy response to Covid–19 protect household incomes?. J. Econ. Inequal..

[66] Warner M.E., Zhang X. (2021). Social safety nets and COVID–19 stay home orders across US states: A comparative policy analysis. J. Comp. Policy Anal. Res. Pract..

[67] Chan J.F.-W., Yuan S., Kok K.-H., To K.K.-W., Chu H., Yang J., Xing F., Liu J., Yip C.C.-Y., Poon R.W.-S. (2020). A familial cluster of pneumonia associated with the 2019 novel coronavirus indicating person–to–person transmission: A study of a family cluster. Lancet.

[68] Whitelaw S., Mamas M.A., Topol E., Van Spall H.G. (2020). Applications of digital technology in COVID–19 pandemic planning and response. Lancet Digit. Health.

[69] Golechha M. (2020). COVID–19 containment in Asia’s largest urban slum Dharavi–Mumbai, India: Lessons for policymakers globally. J. Urban Health.

[70] Kucharski A.J., Klepac P., Conlan A.J., Kissler S.M., Tang M.L., Fry H., Gog J.R., Edmunds W.J., Emery J.C., Medley G. (2020). Effectiveness of isolation, testing, contact tracing, and physical distancing on reducing transmission of SARS–CoV–2 in different settings: A mathematical modelling study. Lancet Infect. Dis..

[71] Lunz D., Batt G., Ruess J. (2021). To quarantine, or not to quarantine: A theoretical framework for disease control via contact tracing. Epidemics.

[72] An B.Y., Tang S.-Y. (2020). Lessons from COVID–19 responses in East Asia: Institutional infrastructure and enduring policy instruments. Am. Rev. Public Adm..

[73] Huang I.Y.F. (2020). Fighting COVID-19 through government initiatives and collaborative governance: The Taiwan experience. Public Adm. Rev..

[74] Lin C., Braund W.E., Auerbach J., Chou J.-H., Teng J.-H., Tu P., Mullen J. (2020). Policy decisions and use of information technology to fight coronavirus disease, Taiwan. Emerg. Infect. Dis..

[75] Moorkamp M., Torenvlied R., Kramer E.H. (2020). Organizational synthesis in transboundary crises: Three principles for managing centralization and coordination in the corona virus crisis response. J. Contingencies Crisis Manag..

[76] Gigliotti R.A. (2016). Leader as performer; leader as human: A discursive and retrospective construction of crisis leadership. Atl. J. Commun..

[77] McGuire D., Cunningham J.E., Reynolds K., Matthews–Smith G. (2020). Beating the virus: An examination of the crisis communication approach taken by New Zealand Prime Minister Jacinda Ardern during the Covid–19 pandemic. Hum. Resour. Dev. Int..

[78] Serikbayeva B., Abdulla K., Oskenbayev Y. (2021). State capacity in responding to COVID–19. Int. J. Public Adm..

[79] Gong B., Zhang S., Yuan L., Chen K.Z. (2020). A balance act: Minimizing economic loss while controlling novel coronavirus pneumonia. J. Chin. Gov..

[80] Hyland–Wood B., Gardner J., Leask J., Ecker U.K. (2021). Toward effective government communication strategies in the era of COVID–19. Humanit. Soc. Sci. Commun..

[81] Agley J. (2020). Assessing changes in US public trust in science amid the COVID–19 pandemic. Public Health.

[82] Mohd Hanafiah K., Ng C., Wan A.M. (2021). Effective Communication at Different Phases of COVID–19 Prevention: Roles, Enablers and Barriers. Viruses.

[83] Roy D., Tripathy S., Kar S.K., Sharma N., Verma S.K., Kaushal V. (2020). Study of knowledge, attitude, anxiety & perceived mental healthcare need in Indian population during COVID–19 pandemic. Asian J. Psychiatry.

[84] Ipsen C., van Veldhoven M., Kirchner K., Hansen J.P. (2021). Six key advantages and disadvantages of working from home in Europe during COVID–19. Int. J. Environ. Res. Public Health.

[85] Drašler V., Bertoncelj J., Korošec M., Pajk Žontar T., Poklar Ulrih N., Cigić B. (2021). Difference in the attitude of students and employees of the university of ljubljana towards work from home and online education: Lessons from COVID–19 pandemic. Sustainability.

[86] Dubey A.D., Tripathi S. (2020). Analysing the sentiments towards work–from–home experience during covid–19 pandemic. J. Innov. Manag..

[87] Kramer A., Kramer K.Z. (2020). The Potential Impact of the COVID–19 Pandemic on Occupational Status, Work from Home, and Occupational Mobility.

[88] Festa G., Kolte A., Carli M.R., Rossi M. (2021). Envisioning the challenges of the pharmaceutical sector in the Indian health–care industry: A scenario analysis. J. Bus. Ind. Mark..

[89] Festa G., Rossi M., Kolte A., Marinelli L. (2020). The contribution of intellectual capital to financial stability in Indian pharmaceutical companies. J. Intellect. Cap..

[90] Tsui A.B., Chan C.K., Harfitt G., Leung P. (2020). Crisis and opportunity in teacher preparation in the pandemic: Exploring the “adjacent possible”. J. Prof. Cap. Community.

[91] Tan M.K.B., Tan C.M. (2021). Curating wellness during a pandemic in Singapore: COVID–19, museums, and digital imagination. Public Health.

[92] Xie X., Siau K., Nah F.F.-H. (2020). COVID–19 pandemic–online education in the new normal and the next normal. J. Inf. Technol. Case Appl. Res..

[93] Bohnsack R., Kolk A., Pinkse J., Bidmon C.M. (2020). Driving the electric bandwagon: The dynamics of incumbents’ sustainable innovation. Bus. Strategy Environ..

[94] Abomhara M., Yayilgan S.Y., Nweke L.O., Székely Z. (2021). A comparison of primary stakeholders’ views on the deployment of biometric technologies in border management: Case study of SMart mobILity at the European land borders. Technol. Soc..

[95] Steffens F. (2020). Facing up to the new world of border control. Biom. Technol. Today.

[96] Janparvar M., Bahrami Jaf S., Shahbazi M., Chatterjee U. (2021). Control and maintenance of borders due to the expansion of the Kurdish ethnic group on both sides of the Iranian–Iraqi border. Geo J..

[97] Faour–Klingbeil D., Osaili T.M., Al–Nabulsi A.A., Jemni M., Todd E.C. (2021). The public perception of food and non–food related risks of infection and trust in the risk communication during COVID–19 crisis: A study on selected countries from the Arab region. Food Control.

[98] Soava G., Mehedintu A., Sterpu M., Grecu E. (2021). The impact of the COVID–19 pandemic on electricity consumption and economic growth in Romania. Energies.

[99] Hsieh C.-W., Wang M., Wong N.W., Ho L.K.-k. (2021). A whole–of–nation approach to COVID–19: Taiwan’s National Epidemic Prevention Team. Int. Political Sci. Rev..

[100] Rouleau J., Gosselin L. (2021). Impacts of the COVID–19 lockdown on energy consumption in a Canadian social housing building. Appl. Energy.

[101] Ullah S., Ahmad N., Khan F.U., Badulescu A., Badulescu D. (2021). Mapping interactions among green innovations barriers in manufacturing industry using hybrid methodology: Insights from a developing country. Int. J. Environ. Res. Public Health.

[102] Bañuls V.A., Turoff M. (2011). Scenario construction via Delphi and cross–impact analysis. Technol. Forecast. Soc. Chang..

[103] Bañuls V.A., Turoff M., Hiltz S.R. (2013). Collaborative scenario modeling in emergency management through cross–impact. Technol. Forecast. Soc. Chang..

[104] Zhang Y., Weng W., Huang Z. (2018). A scenario–based model for earthquake emergency management effectiveness evaluation. Technol. Forecast. Soc. Chang..

[105] Chang M.-S., Tseng Y.-L., Chen J.-W. (2007). A scenario planning approach for the flood emergency logistics preparation problem under uncertainty. Transp. Res. Part E Logist. Transp. Rev..

[106] Wang R., Wang E., Li L., Li W. (2022). Evaluating the Effectiveness of the COVID–19 Emergency Outbreak Prevention and Control Based on CIA–ISM. Int. J. Environ. Res. Public Health.

[107] Jørgensen F., Bor A., Lindholt M.F., Petersen M.B. (2021). Public support for government responses against COVID–19: Assessing levels and predictors in eight Western democracies during 2020. West Eur. Politics.

[108] Coccia M. (2021). The relation between length of lockdown, numbers of infected people and deaths of Covid–19, and economic growth of countries: Lessons learned to cope with future pandemics similar to Covid–19 and to constrain the deterioration of economic system. Sci. Total Environ..

[109] Coccia M. (2021). Effects of the spread of COVID–19 on public health of polluted cities: Results of the first wave for explaining the dejà vu in the second wave of COVID–19 pandemic and epidemics of future vital agents. Environ. Sci. Pollut. Res..

[110] Coccia M. (2021). The effects of atmospheric stability with low wind speed and of air pollution on the accelerated transmission dynamics of COVID–19. Int. J. Environ. Stud..

[111] Coccia M. (2021). High health expenditures and low exposure of population to air pollution as critical factors that can reduce fatality rate in COVID–19 pandemic crisis: A global analysis. Environ. Res..

[112] Sagan A., Thomas S., McKee M., Karanikolos M., Azzopardi–Muscat N., de la Mata I., Figueras J., Organization, W.H. (2020). COVID–19 and health systems resilience: Lessons going forwards. Eurohealth.

[113] Craig G. (2021). Kindness and Control: The Political Leadership of Jacinda Ardern in the Aotearoa New Zealand COVID–19 Media Conferences. J. Media.

[114] Ruben B.D., Gigliotti R.A. (2016). Leadership as social influence: An expanded view of leadership communication theory and practice. J. Leadersh. Organ. Stud..

[115] Shehzad K., Sarfraz M., Shah S.G.M. (2020). The impact of COVID–19 as a necessary evil on air pollution in India during the lockdown. Environ. Pollut..

[116] Zambrano–Monserrate M.A., Ruano M.A. (2020). Has air quality improved in Ecuador during the COVID–19 pandemic? A parametric analysis. Air Qual. Atmos. Health.

[117] Paulson K.R., Kamath A.M., Alam T., Bienhoff K., Abady G.G., Abbas J., Abbasi–Kangevari M., Abbastabar H., Abd–Allah F., Abd–Elsalam S.M. (2021). Global, regional, and national progress towards Sustainable Development Goal 3.2 for neonatal and child health: All–cause and cause–specific mortality findings from the Global Burden of Disease Study 2019. Lancet.

[118] Ullah A., Pinglu C., Ullah S., Abbas H.S.M., Khan S. (2021). The role of e–governance in combating COVID–19 and promoting sustainable development: A comparative study of China and Pakistan. Chin. Political Sci. Rev..

[119] Li Z., Chen Q., Feng L., Rodewald L., Xia Y., Yu H., Zhang R., An Z., Yin W., Chen W. (2020). Active case finding with case management: The key to tackling the COVID–19 pandemic. Lancet.

[120] Vandenberg O., Martiny D., Rochas O., van Belkum A., Kozlakidis Z. (2021). Considerations for diagnostic COVID–19 tests. Nat. Rev. Microbiol..

[121] Flaxman S., Mishra S., Gandy A., Unwin H.J.T., Mellan T.A., Coupland H., Whittaker C., Zhu H., Berah T., Eaton J.W. (2020). Estimating the effects of non–pharmaceutical interventions on COVID–19 in Europe. Nature.

[122] Drake R.E., Sederer L.I., Becker D.R., Bond G.R. (2021). COVID–19, Unemployment, and Behavioral Health Conditions: The Need for Supported Employment. Adm. Policy Ment. Health Ment. Health Serv. Res..

[123] Szentesi S.G., Cuc L.D., Feher A., Cuc P.N. (2021). Does COVID–19 Affect Safety and Security Perception in the Hospitality Industry? A Romanian Case Study. Sustainability.

[124] Eichenbaum M.S., Rebelo S., Trabandt M. (2021). The macroeconomics of epidemics. Rev. Financ. Stud..

[125] Ferguson N., Laydon D., Nedjati–Gilani G., Imai N., Ainslie K., Baguelin M., Bhatia S., Boonyasiri A., Cucunubá Z., Cuomo–Dannenburg G. (2020). Report 9: Impact of non–pharmaceutical interventions (NPIs) to reduce COVID19 mortality and healthcare demand. Imp. Coll. Lond..

[126] Chesbrough H. (2020). To recover faster from Covid–19, open up: Managerial implications from an open innovation perspective. Ind. Mark. Manag..

[127] Bogoch I.I., Watts A., Thomas–Bachli A., Huber C., Kraemer M.U., Khan K. (2020). Pneumonia of unknown aetiology in Wuhan, China: Potential for international spread via commercial air travel. J. Travel Med..

[128] Thu T.P.B., Ngoc P.N.H., Hai N.M. (2020). Effect of the social distancing measures on the spread of COVID–19 in 10 highly infected countries. Sci. Total Environ..

[129] Anderson R.M., Heesterbeek H., Klinkenberg D., Hollingsworth T.D. (2020). How will country–based mitigation measures influence the course of the COVID–19 epidemic?. Lancet.

[130] Qiu Y., Chen X., Shi W. (2020). Impacts of social and economic factors on the transmission of coronavirus disease 2019 (COVID–19) in China. J. Popul. Econ..

[131] Das D., Datta A., Kumar P., Kazancoglu Y., Ram M. (2021). Building supply chain resilience in the era of COVID–19: An AHP–DEMATEL approach. Oper. Manag. Res..

[132] ALESSA A.A., Alotaibie T.M., Elmoez Z., Alhamad H.E. (2021). Impact of COVID–19 on Entrepreneurship and Consumer Behaviour: A Case Study in Saudi Arabia. J. Asian Financ. Econ. Bus..

[133] Geirdal A.Ø., Ruffolo M., Leung J., Thygesen H., Price D., Bonsaksen T., Schoultz M. (2021). Mental health, quality of life, wellbeing, loneliness and use of social media in a time of social distancing during the COVID–19 outbreak. A cross–country comparative study. J. Ment. Health.

[134] Howard M.C. (2020). Understanding face mask use to prevent coronavirus and other illnesses: Development of a multidimensional face mask perceptions scale. Br. J. Health Psychol..

[135] Crayne M.P. (2020). The traumatic impact of job loss and job search in the aftermath of COVID–19. Psychol. Trauma Theory Res. Pract. Policy.

[136] Smith R.D. (2006). Responding to global infectious disease outbreaks: Lessons from SARS on the role of risk perception, communication and management. Soc. Sci. Med..

[137] Abbas J., Wang D., Su Z., Ziapour A. (2021). The role of social media in the advent of COVID–19 pandemic: Crisis management, mental health challenges and implications. Risk Manag. Healthc. Policy.

[138] Abdou A.M. (2021). Good governance and COVID-19: The digital bureaucracy to response the pandemic (Singapore as a model). J. Public Aff..

[139] Dirani K.M., Abadi M., Alizadeh A., Barhate B., Garza R.C., Gunasekara N., Ibrahim G., Majzun Z. (2020). Leadership competencies and the essential role of human resource development in times of crisis: A response to COVID–19 pandemic. Hum. Resour. Dev. Int..

[140] Sanchez–Duque J.A., Orozco–Hernandez J.P., Marin–Medina D.S., Arteaga–Livias K., Pecho–Silva S., Rodriguez–Morales A.J., Dhama K. (2020). Economy or health, constant dilemma in times of pandemic: The case of coronavirus disease 2019 (COVID–19). J. Pure Appl. Microbiol..

[141] Ather A., Patel B., Ruparel N.B., Diogenes A., Hargreaves K.M. (2020). Coronavirus disease 19 (COVID–19): Implications for clinical dental care. J. Endod..

[142] Martin-Howard S., Farmbry K. (2020). Framing a needed discourse on health disparities and social inequities: Drawing lessons from a pandemic. Public Adm. Rev..

[143] Bonnet F., Ehmke E., Hagemejer K. (2010). Social security in times of crisis. Int. Soc. Secur. Rev..

[144] Rane S.B., Kirkire M.S. (2016). Analysis of barriers to medical device development in India: An interpretive structural modelling approach. Int. J. Syst. Assur. Eng. Manag..

[145] Ullah S., Khan F.U., Ahmad N. (2022). Promoting sustainability through green innovation adoption: A case of manufacturing industry. Environ. Sci. Pollut. Res..

[146] Talib F., Rahman Z., Qureshi M. (2011). An interpretive structural modelling approach for modelling the practices of total quality management in service sector. Int. J. Model. Oper. Manag..

[147] Gupta A., Gupta S., Shekhar (2021). Determining Interrelationship Between Factors Impacting Foreign Direct Investment in Tourism: An ISM–based Approach. Asia–Pac. J. Manag. Res. Innov..

[148] Khaba S., Bhar C. (2017). Modeling the key barriers to lean construction using interpretive structural modeling. J. Model. Manag..

